# Secretoglobin Felis Domesticus 1 (Fel d 1) specific immunoglobulin E in diagnosis of patients with respiratory allergy to cats

**DOI:** 10.1038/s41598-026-42206-7

**Published:** 2026-03-24

**Authors:** Nermine Yehia Moawad Alshenawy, Manal Zaghloul Mahran, Marwa Rushdy ElNajjar, Mai Asem Ali AlBdry, Zeinab Ahmed Ashour, Lamyaa Salem

**Affiliations:** 1 Department of Clinical Pathology, Faculty of Medicine, Ain Shams University, Cairo, Egypt; 2 Department of Internal Medicine, Faculty of Medicine, Ain Shams University, Cairo, Egypt

**Keywords:** Cat allergy, Fel d 1, Respiratory allergy, Biomarkers, Diseases, Immunology, Medical research

## Abstract

Cat allergy is a common cause of respiratory allergic diseases, such as asthma and allergic rhinitis. Secretoglobin Felis domesticus 1 (Fel d 1) is the major cat allergen, widely distributed in the environment and difficult to avoid. Measuring Fel d 1-specific Immunoglobulin E (sIgE) provides an important diagnostic approach for evaluating sensitisation and disease severity. The present study assessed the diagnostic value of allergen-specific IgE to secretoglobin Fel d 1 in Egyptian adult patients with respiratory allergy sensitised to cats. This cross-sectional study included 35 patients with clinical diagnoses of asthma and/or allergic rhinitis, all positive for cat dander by skin prick test (SPT). Complete blood count, total IgE, and serum Fel d 1-sIgE (measured using ImmunoCAP) were performed. Of the 35 patients, 40% tested positive for Fel d 1-sIgE. Positive Fel d1 -sIgE was significantly associated with older age, history of cat exposure, and family history of allergy (*p* < 0.05). Patients with positive Fel d 1-sIgE levels showed significantly higher total IgE, leukocyte, and eosinophil counts. Fel d 1-sIgE levels were positively correlated with asthma and allergic rhinitis severity. ImmunoCAP Fel d 1-sIgE testing accurately differentiates true cat allergy from cross-sensitization. This study represents one of the first regional data utilizing this method in an Egyptian cohort, enhancing diagnostic precision, assessing disease severity, and supporting personalized treatment strategies.

## Introduction

One of the IgE-mediated allergic diseases is allergic rhinitis (AR) which is triggered by airborne allergens that contact the nasal mucosa of allergic individuals. Common clinical manifestations include sneezing, rhinorrhea, and nasal obstruction, which are often accompanied by ocular symptoms^1^. AR significantly impairs health-related quality of life, particularly sleep quality, productivity, and overall work performance^2^.

Allergic rhinitis can coexist with asthma, which is another allergic disease of the respiratory tract. Asthma causes airflow limitation and symptoms, including shortness of breath, cough and chest tightness^3^. Recent studies indicate that the coexistence of rhinitis and asthma may represent a facet of a systemic airway disease impacting the entire respiratory tract, referred to as respiratory allergies^4^.

Feline-derived inhalant allergens are a prominent cause of respiratory allergies, with cat allergies identified as the most prevalent immunoglobulin (Ig) E-mediated hypersensitivity. Cat allergies are believed to affect almost one in five individuals worldwide^5^.

The major cat allergen, Secretoglobin Fel d 1, is highly pervasive and has been detected in dust samples from both cat- and non-cat owning households. It has also been found in public places such as schools and offices, which makes the concept of allergen avoidance very challenging^6^.

Secretoglobin Fel d 1 is produced by cats’ salivary, lacrimal, and sebaceous glands, as well as their anal sacs. It is also frequently found in skin and hair of felines. Secretoglobin Fel d 1 is dispersed on cats’ fur during grooming and sheds with hair and dander^7^.

The application of molecular-based techniques such as ImmunoCAP, for cat allergy diagnosis includes the use of the major cat allergen, namely Secretoglobin Fel d, as well as cross-reacting allergen components, taking into account molecules that may impede allergy diagnosis in vitro. Identifying different molecular cat allergens with medical relevance has led to the development of several singleplex and multiplex molecular IgE immunoassays that allow for a more accurate diagnosis of patients with cat allergy^8^.

The management of cat allergies requires a multimodal approach. One of the key lines of treatment that is guideline-approved for cat allergy is allergen immunotherapy (AIT), which can change the disease’s natural course and have a long-term impact on symptoms^9^. It has been shown that the clinical success of subcutaneous cat immunotherapy was linked to high levels of major allergens in cat allergen extracts^10^.

While cat allergy is a recognized clinical problem in Egypt, only a limited number of published studies to date have utilized ImmunoCAP for its diagnosis, and even fewer have explicitly focused on the major cat allergen, Secretoglobin Fel d 1.

### Aim of the work

To assess the use of allergen-specific IgE to secretoglobin Fel d 1 in the diagnosis of respiratory allergy among adult Egyptian patients sensitised to cats.

### Subjects and methods

This cross-sectional study was conducted on a stratified random sample of 35 patients diagnosed with respiratory allergies recruited from the outpatient clinic of the Internal Medicine Allergy and Immunology Department of Ain Shams University Hospitals between April 2024 and September 2024. Laboratory work was performed at the Clinical Pathology Department of Ain Shams University Hospitals. All participants provided written informed consent prior to enrolment in the study, according to the regulations of the Ethical Committee of the Faculty of Medicine, Ain Shams University (MS FWA 000017585/ MS 701/2023).

The inclusion criteria for our study included adult male and female patients, all of whom were positive for cat dander by skin prick test (SPT). Patients with asthma were diagnosed according to the Global Initiative for Asthma (GINA, 2025)^3^, and those with AR were diagnosed according to allergic rhinitis and its impact on asthma guidelines report (ARIA, 2022)^11^. The exclusion criteria included patients receiving corticosteroids, oral antihistamines, or any other drugs that interfere with SPT results; patients with autoimmune diseases; patients who were previously or currently immunized with allergen-specific immunotherapy; pregnant and lactating women; patients with dermographism or chronic eczema; patients with severe asthma; and severe cardiovascular comorbidity.

All recruited patients underwent detailed history taking regarding demographic data, age, gender, address, possible triggers of cat allergy, family history of allergy and history of allergic disease, including onset, course, duration, severity, frequency of attacks, and disease control. Moreover, patients underwent thorough clinical examinations to assess the concurrent status of illness and confirm the exclusion criteria, emphasising the signs and symptoms of respiratory allergy.

Skin prick tests (SPT) were performed for all patients using standardized allergen extracts from cat dander, in addition to common inhalants and food allergens provided by Hamilton (Omega, Allergy OVERSEAS consultants Inc., Canada). The test was performed according to the guidelines for the chemical diagnosis of allergic diseases^12^. The test procedures were explained to the patients after obtaining informed consent. All antihistamines were stopped 5–7 days before performing the test. The test was performed on the volar aspect of each participant’s forearm. The skin was punctured using a calibrated lancet (1 mm) held vertically while introducing a drop of diluted purified allergen. Allergen extracts involved in the test, in addition to cat dander included common environment aeroallergens such as Candida, Penicillium, mixed moulds, house dust mites (*Dermatophagoides farinae*, *Dermatophagoides pteronyssoides*), dust, straw, Timothy grass, birch, cockroach, pigeon feather, latex, tobacco, straw and extract from mixed pollens. The food allergens included fish, eggs, milk, mango, chocolate, Diet C (low chemical diet) and peanuts. A drop of histamine (10 mg/ml) and saline solution was used as a positive and negative control, respectively. The maximum or mean diameter of the wheal for various allergens was measured after 20 min. A wheal of 3 mm or more in diameter was considered positive, indicating sensitization to that allergen. A positive SPT for cats and other tested allergens defined polysensitisation.

In reference to laboratory investigations, two mL of venous blood was collected aseptically in tri-potassium ethylene diamine tetra-acetic acid (K3 EDTA) vacutainer tubes to perform CBC with differential count using a Coulter counter (T660; Beckman Coulter, Brea, CA, USA). Another five ml of venous blood was collected by venipuncture from each participant into gel vacutainer tubes (Becton Dickinson, Oxford, UK). Blood was allowed to clot, and serum was separated by centrifugation at 3500 rpm for 10 min and then stored in aliquots at − 20 °C until used for measurement of total IgE levels by ELISA (DRG ELISA kit, REF EIA-1788, GmbH, Germany) and measurement of Fel d 1 sIgE using ImmunoCAP; repeated freezing and thawing of serum samples was avoided.

ImmunoCAP Fel d 1 sIgE measurements were performed using Phadia 250 (Thermo Fisher Scientific, Uppsala, Sweden). This technique relies on a fluoroenzyme immunoassay (FEIA) to objectively detect circulating sIgE antibodies and sensitisation to specific allergens.

### Interpretation

The results were provided in terms of specific IgE antibody concentration, expressed in kilo units of allergen–specific IgE per liter (kU_A_/L).

Values equal to or exceeding 0.35 kU_A_/L indicate specific IgE antibodies to one of the single ImmunoCAP allergen components.

A value less than 0.35 kU_A_/L indicates undetectable or very low levels of allergen-specific IgE antibodies.

The investigators performing and interpreting SPT and ImmunoCAP results were blinded to clinical severity scores, to reduce potential observer bias.

While the Phadia ImmunoCAP system is recognized globally as the gold standard for in vitro allergy diagnostics, its application for Fel d 1 molecular testing in Egypt remains undocumented. To the best of our knowledge, our study conducted at Ain Shams University is one of the initial studies to evaluate Fel d 1 in an Egyptian cohort.

### Statistical analysis

Statistical analyses were performed using the 27th version of the Statistical Package for the Social Sciences (SPSS) (IBM Corp., Armonk, NY, USA) and Excel (Microsoft Office 2010). Quantitative data were described using mean, standard deviation, and range when parametric and median, interquartile range (IQR) when data were found to be non-parametric. The choice of non-parametric tests was justified given the small sample size and data distribution. Qualitative variables were described using numbers and percentages. The p-value was deemed significant as follows: p-values > 0.05 were non-significant (NS), p-values < 0.05 were significant (S), and p-values < 0.01 were highly significant (HS).

## Results

This cross-sectional study included 35 Egyptian patients provisionally diagnosed with cat allergy who attended the outpatient clinic of the Allergy & Immunology Department, Ain Shams University Hospitals. The main demographic characteristics of the studied patients are presented in Table 1.

**Table 1:** Demographic data and characteristics of the patients.

		Cases (No. = 35)
Gender	Male	8 (22.9%)
	Female	27 (77.1%)
Age (Years)	Mean ± SD	29.4 ± 4.53
	Range	21–38
Exposure to cats	No	19 (54.3%)
	Yes	16 (45.7%)
Residence	Cairo	20 (57.1%)
	Other regions	15 (42.9%)
Family history of allergy	Negative	31 (88.6%)
	Positive	4 (11.4%)

The most common allergens to which the patients were exposed were mango and eggs, and the lowest percentage was recorded for chocolate and peanuts.

**Fig. 1:**
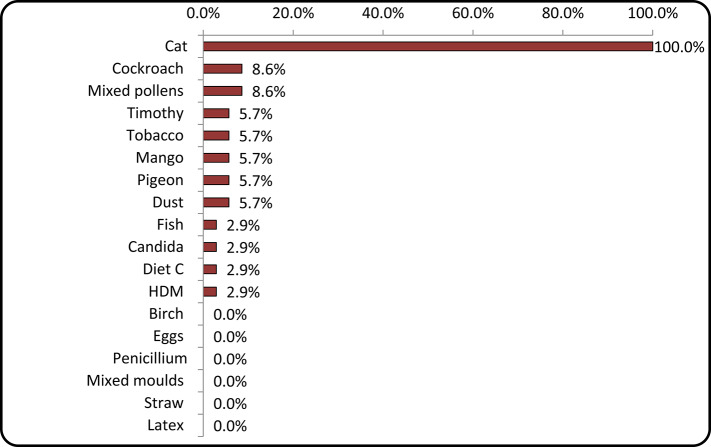
Horizontal bar chart representing the percentage of positive SPT among patients in our study.

Regarding SPT, all patients were positive for cat dander, followed by other allergens, including cockroach, tobacco, mango, and house dust mite (HDM). No incidence of allergy to birch, straw, or latex was recorded (Fig. 1).

Patients in the study presented clinically with skin manifestations, 22 patients (62.9%), asthma 18 patients (51.4%), or AR 18 patients (51.4%). Twenty-two patients (62.9%) showed seasonal variations in their symptoms, whereas 13 patients (37.1%) did not. Nine patients (25.7%) responded to AIT, whereas 26 (74.3%) did not. The laboratory investigations performed on the patients are presented in Table 2.

**Table 2:** Descriptive data of laboratory parameters.

		No. = 35
Total IgE (IU/mL)	Median (IQR)	212 (161–333)
	Range	112–800
TLC (10^3^/mm^3^)	Mean ± SD	6.9 ± 2.08
	Range	3.7–12.1
Eosinophil (10^3^/mm^3^)	Median (IQR)	0.2 (0.1–0.4)
	Range	0.01–1
Platelets (10^3^/mm^3^)	Mean ± SD	247.91 ± 59.94
	Range	157–406
Fel d 1 (KU_A_/L)	Median (IQR) Range	0.01 (0–2.89) 0–20.9
	Negative Positive	21 (60%) 14 (40%)
Class of classification	Class 0 Class 1 Class 2 Class 4	21 (60%) 1 (2.85%) 10 (28.57%) 3 (8.57%)

IgE: Immunoglobulin E, TLC: total leukocyte count.

### Comparative statistics among the studied patients

A statistically significant difference in age, history of exposure to cats, residence, and family history of cat allergy was found between negative and positive Fel d 1 patients (Table 3).

**Table 3:** Comparison between negative and positive Fel d 1 patients regarding demographic data and characteristics.

		Fel d 1		Test value	P-value	Sig.
		Negative	Positive			
		No. = 20	No. = 15			
Gender	Male	4 (20.0%)	4 (26.7%)	0.216*	0.642	NS
	Female	16 (80.0%)	11 (73.3%)			
Age (years)	Mean ± SD	27.70 ± 3.69	31.67 ± 4.67	-2.810•	0.008	HS
	Range	21–33	22–38			
Exposure to cats	No	17 (85.0%)	2 (13.3%)	17.740*	0.000	HS
	Yes	3 (15.0%)	13 (86.7%)			
Residence	Cairo	18 (90.0%)	2 (13.3%)	20.572*	0.000	HS
	Other regions	2 (10.0%)	13 (86.7%)			
Family history of cat allergy	Negative	20 (100.0%)	11 (73.3%)	6.022*	0.014	S
	Positive	0 (0.0%)	4 (26.7%)			
Occupation	HW - Retired person Unskilled worker Employee Professional	16 (80.0%) 2 (10.0%) 1 (5.0%) 1 (5.0%)	11 (73.3%)	0.216*	0.975	NS
			2 (13.3%) 1 (6.7%) 1 (6.7%)			
Onset of allergic manifestations	Recent (months)	20 (100.0%)	2 (13.3%)	27.576*	0.000	HS
	Years	0 (0.0%)	13 (86.7%)			

P-value > 0.05: Non-significant; P-value < 0.05: Significant; p-value < 0.01: Highly significant.

*Chi-square test; •Independent t-test.

HS: Highly significant, NS: Non-significant, HW: housewife.

There was a statistically significant increase in total IgE levels in patients with positive Fel d 1 compared to those with negative Fel d 1. In addition, the TLC and eosinophil counts were significantly higher in the positive Fel d 1 group than in the negative Fel d 1 group (Table 4).

**Table 4:** Comparison between negative and positive Fel d 1 groups regarding laboratory investigations of the studied patients.

		Fel d 1		Test value	P-value	Sig.
		Negative	Positive			
		No. = 20	No. = 15			
Total IgE (IU/ml)	Median (IQR)	194 (163–245)	360 (161–468)	-2.250≠	0.024	S
	Range	112–312	112–800			
TLC (10^3^/mm^3^)	Mean ± SD	5.93 ± 1.45	8.19 ± 2.12	-3.748•	0.001	HS
	Range	3.7–8.5	5.9–12.1			
Eosinophil (10^3^/mm^3^)	Median (IQR)	0.1 (0.05–0.2)	0.4 (0.1–0.5)	-2.076≠	0.038	S
	Range	0.01–1	0.01–0.6			
Platelet (10^3^/mm^3^)	Mean ± SD	231.45 ± 49.39	269.87 ± 67.16	-1.952•	0.059	NS
	Range	157–315	166–406			

P-value > 0.05: Non significant; P-value < 0.05: Significant; P-value < 0.01: Highly significant •: Independent t-test; ≠: Mann-Whitney test.

IgE: Immunoglobulin E, TLC: total leukocyte count.

### Correlation statistics among the studied groups

Fel d 1 levels were positively correlated with age, total IgE, TLC, and eosinophil count, but there was no statistically significant correlation between Fel d 1 levels and platelet counts among the patients under study (Table 5).

**Table 5:** Correlation of Fel d 1 levels with other studied parameters among the studied patients.

	Fel d 1 (KU_A_/L)	
	*R*	*P*-value
Age (years)	0.461**	0.005
Total IgE (IU/ml)	0.407*	0.015
TLC (10^3^/mm^3^)	0.395*	0.019
Eosinophil (10^3^/mm^3^)	0.417*	0.013
Platelet (10^3^/mm^3^)	0.145	0.405

*Significant; **Highly significant.

Spearman correlation coefficient.

IgE: Immunoglobulin E, TLC: total leukocyte count.

We performed relationship studies between Fel d1 levels and SPT and clinical presentations of our patients and found that Fel d 1 levels were significantly higher in patients with positive SPT to tobacco and positive SPT to mixed pollen than in patients with a negative SPT to these allergens.

There was a statistically significant difference in Fel d 1 levels between patients with and without asthma. Fel d 1 levels significantly increased with increasing asthma severity. Furthermore, there was a statistically significant increase in the level of Fel d 1 in patients with cat allergy with AR, and Fel d 1 significantly increased with the AR severity (Table 6).

**Table 6:** Relation of Fel d 1 with (SPT) and clinical manifestations among the studied patients.

		Fel d 1 (KU_A_/L)		Test value	P-value	Sig.
		Median (IQR)	Range			
Timothy	Negative	0.01 (0–2.68)	0–20.9	-0.113•	0.910	NS
	Positive	1.5 (0–3)	0–3			
Tobacco	Negative	0 (0–2.49)	0–20.3	-2.493•	0.013	S
	Positive	20.75 (20.6–20.9)	20.6–20.9			
Fish	Negative	0.01 (0–2.89)	0–20.9	-0.421•	0.674	NS
	Positive	0.36 (0.36–0.36)	0.36–0.36			
Mango	Negative	0 (0–2.68)	0–20.6	-1.737•	0.082	NS
	Positive	11.5 (2.11–20.9)	2.11–20.9			
Candida	Negative	0.01 (0–2.89)	0–20.9	-0.947•	0.344	NS
	Positive	0 (0–0)	0–0			
Diet C	Negative	0.01 (0–2.89)	0–20.9	-0.737•	0.461	NS
	Positive	2.49 (2.49–2.49)	2.49–2.49			
Cockroach	Negative	0.01 (0–2.79)	0–20.9	-0.470•	0.639	NS
	Positive	0 (0–3)	0–3			
Mixed pollens	Negative	0 (0–2.59)	0–20.6	-2.161•	0.031	S
	Positive	20.3 (0.02–20.9)	0.02–20.9			
HDM	Negative	0.01 (0–2.89)	0–20.9	-0.947•	0.344	NS
	Positive	0 (0–0)	0–0			
Pigeon	Negative	0.01 (0–2.68)	0–20.6	-0.604•	0.546	NS
	Positive	10.45 (0–20.9)	0–20.9			
Dust	Negative	0.01 (0–2.89)	0–20.9	-1.360•	0.174	NS
	Positive	0 (0–0)	0–0			
Skin manifestations	Negative	0 (0–0.02)	0–20.6	-1.342•	0.179	NS
	Positive	1.52 (0–3)	0–20.9			
Degree of severity	No	0 (0–0.02)	0–20.6	6.002≠	0.112	NS
	Mild	0.93 (0–2.68)	0–3			
	Moderate	0 (0–3.03)	0–20.3			
	Severe	12 (3.09–20.9)	3.09–20.9			
Asthma	Negative	0 (0–0.01)	0–0.36	-3.438•	0.001	HS
	Positive	2.79 (0–3.09)	0–20.9			
Degree of severity	No	0 (0–0.01)	0–0.36	17.505≠	0.001	HS
	Mild	2.3 (0–2.95)	0–3.17			
	Moderate	3 (0.93–3.03)	0.93–3.03			
	Severe	20.6 (20.3–20.9)	20.3–20.9			
Allergic rhinitis (AR)	Negative	0 (0–0.01)	0–0.36	-3.438•	0.001	HS
	Positive	2.79 (0–3.09)	0–20.9			
Degree of severity	No	0 (0–0.01)	0–0.36	16.617≠	0.001	HS
	Mild	2.49 (0–3)	0–3.17			
	Moderate	1.97 (0.93–3)	0.93–3			
	Severe	20.6 (20.3–20.9)	20.3–20.9			

p-value > 0.05: Non-significant; p-value < 0.05: Significant; P-value < 0.01: Highly significant.

•Mann-Whitney test; ≠Kruskal-Wallis test.

AR: Allergic rhinitis, HDM: House Dust Mites, HS: Highly significant, NS: Non-significant.

## Discussion

Allergy to furry animals remains a prevalent cause of IgE-mediated respiratory disease, with cats and dogs being the most frequent sensitization sources. In Cat allergies, Fel d 1 is the primary allergen responsible for sensitisation^13^. Fel d 1 is a small, stable protein produced by cat sebaceous and salivary glands that spreads into the environment through cat fur and dander. Due to its ubiquity, individuals may develop allergic reactions even without direct exposure to cats, complicating clinical assessment and necessitating precise diagnostic strategies alongside targeted treatment approaches^14^.

Recent diagnostic reviews have emphasised the growing role of component-resolved diagnostics (CRD) in the evaluation of furry animal allergy. By measuring IgE responses to individual molecular allergens rather than whole extracts, CRD improves specificity and helps distinguish genuine sensitization from cross-reactivity, particularly in polysensitised patients^15,16^. The identification of sensitization to major allergens such as Fel d 1 is consistently associated with clinically relevant diseases and supports more accurate patient stratification^14^.

This study aimed to assess the use of allergen-specific IgE to secretoglobin Fel d 1 in the diagnosis of respiratory allergy among adult Egyptian patients sensitised to cats. The study population consisted of 35 Egyptian patients, predominantly females (77.1%), with an age range of 21–38 years (mean age: 29.4 years). The predominance of allergic conditions in females was explained by *Jensen-Jarolim et al.*^17^, who suggested that hormonal and immunological differences may influence the prevalence of allergic diseases. In our study, approximately 45.7% of participants reported direct exposure to cats, while the remaining 54.3% had no known exposure but still exhibited allergic symptoms.

While our findings agree with those of *Riabova et al.*^18^ in showing elevated Fel d 1 levels in asthma, *Custovic et al.*^19^ observed tolerance in high-exposure settings, suggesting that exposure intensity may determine immune outcomes and that high Fel d 1 exposure may, in some cases, paradoxically promote immunological tolerance rather than sensitisation, a factor not addressed in our cohort, raising further questions about the mechanisms underlying sensitisation. This phenomenon is believed to result from mechanisms such as the induction of allergen-specific regulatory T cells, modulation of Th1/Th2 balance, and establishment of mucosal immune tolerance through repeated exposure^20^. The differences in findings across studies highlight the complex interplay between environmental exposure, immune responses, and genetic predispositions in cat-allergic patients, and may also be attributed to variations in population characteristics or methodological approaches.

The diagnostic workup for cat allergy begins with a SPT to detect sensitisation of the individual to cat allergens. In our study, all 35 patients had positive SPT results for cat dander; however, only 14 of them had Fel d 1-specific IgE detected by ImmunoCAP meaning 60% of our Egyptian cohort may not actually require intensive cat-specific interventions.These findings highlight a critical diagnostic gap and is key that traditional SPT often shows high false positivity either due to the sticky nature of cat allergens or cross-reactivity.

Our study underscores the importance of relying on CRD in conjunction with clinical history and SPT to improve the diagnostic accuracy of cat allergy, potentially saving patients from unnecessary costs and lifestyle changes. By applying this technology in Egypt, our study adds evidence from an underrepresented population that aligns local clinical practice with the latest EAACI Molecular Allergology guidelines^21^.

In our study, SPT showed 100% positivity for cat allergens, confirming that all the study participants had some degree of sensitisation. Interestingly, pollen (8.6%), timothy grass (5.7%), and tobacco (5.7%) were additional sensitisers, suggesting that a subset of patients had multiple allergic triggers.

In the current study, seasonal variation in symptoms was reported by 62.9% of patients, a crucial finding that supports the interplay between seasonal pollen exposure and cat allergen-induced respiratory symptoms, which aligns with *Custovic et al.*^19^, who emphasised that pollen exposure can exacerbate allergic symptoms, creating a cumulative allergenic load that worsens cat allergy symptoms during peak pollen seasons.

In support of our findings, *Elkady and Atef*^22^ reported that pollens, including birch and grass pollens, were among the most prevalent aeroallergens in their cohort of AR patients. Although their study did not directly assess the seasonal variation in symptoms or explore the interaction between pollen exposure and cat allergen-induced respiratory symptoms, they acknowledged that the prevalence of aeroallergens is influenced by seasonal and climatic changes. This observation is consistent with our finding that 62.9% of patients reported seasonal fluctuations in symptom severity. Our results are consistent with those of *Custovic et al.*^19^, who highlighted the role of cumulative allergenic load in exacerbating allergic symptoms, particularly during peak pollen seasons. Therefore, our study highlights the temporal dimension of symptom severity in cat-allergic individuals, an aspect that has not been thoroughly examined in regional studies.

Fel d 1-specific IgE levels measured using ImmunoCAP were classified into different classes: class 0 (< 0.35cKU_A_/L), class 1 (0.35–0.7 KU_A_/L), class 2 (0.7–3.5 KU_A_/L), class 3 (3.5–17.5 KU_A_/L), class 4 (17.5–50 KU_A_/L), class 5 (50–100 KU_A_/L), and class 6 (> 100 KU_A_/L). A specific threshold of > 0.35 kU/L is a standard indicator for sensitisation. In our study, 40% of the patients showed positive results for Fel d 1 specific IgE, with the remaining 60% testing negative. Among our Fel d 1-specific IgE-positive cases, the majority (28.57%) fell into class 2, indicating a moderate level of sensitisation, while 8.57% fell into class 4, suggesting a high level of Fel d 1-specific IgE. These findings emphasise that Fel d 1-specific IgE positivity does not always correlate with exposure history, reinforcing the importance of specific IgE testing rather than relying on self-reported exposure. *Riabova et al.*^18^ demonstrated that Fel d 1-specific IgE reactivity varies significantly among allergic individuals, with some highly sensitised patients lacking direct cat exposure. *Trifonova et al.*^23^ found that patients with respiratory symptoms due to cat allergens often exhibit varying degrees of Fel d 1-specific IgE, with a subset showing high levels (class 4 or above), supporting the observed distribution in our study.

The co-existence of asthma and AR was noted in 51.4% of our patients, reinforcing the concept of “united airway disease” where upper and lower respiratory tract inflammations are closely linked. The concept of united airway disease was supported by the findings of *Moghtaderi et al.*^24^. The severity of asthma in our patients was graded as mild (34.3%), moderate (8.6%), and severe (8.6%), with similar distributions observed in AR cases. According to *Sparkes*^13^, cat owners exhibited significantly higher symptom scores than non-cat owners. Cat owners had a greater risk of moderate or severe AR that remained even after therapy (31.8%) than non-cat owners (17.8%).

In our study, skin manifestations were present in 62.9% of patients, with severity classified as mild (25.7%), moderate (31.4%), and severe (5.7%). This high prevalence is in line with the findings of *Kaya et al.*^25^, where 62.9% of children sensitised to cats were symptomatic on exposure, and 15.7% had skin changes (urticaria, erythema, or atopic dermatitis) among those symptomatic patients.

Total IgE levels were elevated in our study group, with a median of 212 IU/mL (range: 112–800 IU/mL). Patients with positive Fel d 1-specific IgE levels showed significantly higher total IgE levels compared to negative cases (*p* = 0.024). *Riabova et al.*^18^ reported similar results.

Total leukocyte count (TLC) and eosinophil count were significantly higher in Fel d 1-positive patients in our study (*p* = 0.001 and *p* = 0.038, respectively), with weak-to-moderate positive correlations between Fel d 1-specific IgE levels and TLC (*r* = 0.395) and eosinophil count (*r* = 0.417). *Trifonova et al.*^23^ similarly noted that eosinophil counts were elevated in cat-allergic patients, further supporting the link between Fel d 1 sensitisation and increased immune activation. This elevation in immune cell parameters suggests a systemic inflammatory response associated with Fel d 1 sensitisation. The observed increase in eosinophil counts among Fel d 1-positive individuals supports the hypothesis that sensitisation to this major cat allergen is associated with enhanced eosinophilic activity, reflecting a Th2-mediated immune response, emphasising IL-5-driven eosinophil recruitment in sensitised individuals^26^.

Fel d 1 levels were significantly higher in patients sensitised to tobacco and mixed pollens. This suggests that co-exposure to cigarette smoke and pollen allergens may amplify allergic responses to cat allergens, worsening respiratory symptoms.

While studies like that of *Johansson et al.*^27^ suggest that tobacco smoke and pollen exposure may potentiate allergic responses to Fel d 1, other studies, such as that of *Trifonova et al.*^23^, indicate that not all sensitised individuals develop severe symptoms, suggesting the influence of genetic and environmental factors in modulating immune responses. These findings collectively emphasise the role of Fel d 1 as a potential indicator of disease severity while acknowledging the variability in allergic responses based on co-exposures and individual susceptibility. Recent evidence confirms that elevated levels of Fel d 1-sIgE are not only diagnostic but also significant prognostic markers for type 2 airway inflammation and the progression from allergic rhinitis to severe asthma^28^.

The current study reported various food allergens in the patients’ history, including mango (28.6%) and eggs (28.6%), followed by milk (25.7%) and medications (22.9%). *Popescu et al.*^8^ highlighted that cross-reactivity between food and inhalant allergens is common in allergic patients, particularly with proteins found in eggs, milk, and plant-derived foods such as mango. Oral Allergy Syndrome (OAS), also known as pollen-food allergy syndrome, is an IgE-mediated hypersensitivity reaction that occurs in individuals sensitised to pollen allergens who subsequently react to structurally similar proteins found in certain fresh fruits, vegetables, and nuts. This condition results from cross-reactivity between homologous proteins in pollen (such as birch, grass, or ragweed) and plant-derived foods. Clinically, OAS is distinguished by the sudden onset of itching, tingling, or slight swelling of the lips, tongue, palate, and throat following the consumption of raw fruits or vegetables^29^.

The management of allergies to furry animals including cat allergies, relies on a combination of allergen avoidance, pharmacotherapy, and AIT. While complete avoidance is often impractical, pharmacological treatment remains the cornerstone of symptom control. Allergen immunotherapy is currently the only disease-modifying option and should be considered in patients with persistent symptoms and confirmed clinically relevant sensitization^30^.

In our study, only 25.7% of the patients had a positive response to AIT, whereas 74.3% were non-responders. This low response rate to AIT could be attributed to factors such as long-standing disease duration or insufficient immunotherapy dosage.

The low response rate to AIT in our study (25.7%) was consistent with findings from *Jensen-Jarolim et al.*^17^ and *Trifonova et al.*^23^, who reported that Fel d 1-specific immunotherapy can be less effective in cases of high environmental exposure, where persistent allergen presence limits immune system modulation.

However, some studies contradict the low AIT response rate; *Riabova et al.*^18^ found that up to 50% of cat-allergic patients showed significant improvement with AIT, particularly those who received early intervention with well-optimized doses. This suggests that treatment efficacy may be influenced by factors such as timing, patient selection, and adherence to therapy, highlighting the need for individualized treatment approaches in cat allergy management.

Incorporating Fel d 1-sIgE into the management of Egyptian patients could aid in refined immunotherapy selection where AIT is prioritized for Fel d 1-monosensitized patients, who typically show the most robust clinical response. Since Fel d 1 is a “sticky” allergen that persists for months, measuring specific IgE helps clinicians advise patients on the necessity of aggressive environmental controls versus simpler interventions^15^. Moreover, accurate Fel d 1 profiling could have potential clinical applications in emerging management strategies, such as anti-Fel d 1 IgY-enriched diets for cats, which have been recently shown to significantly reduce human symptoms in home settings as stated by *Colosimo et al.*^28^. Another study by *Bousquet et al.*^31^. also showed that reducing Fel d 1 at the source (via cat diet) significantly improves global allergy symptoms and work productivity. Additionally, Hedrick et al.^32^ confirmed the long-term safety and efficacy of anti-Fel d 1 management strategies, supporting their role in integrated care pathways.

One of the notable strengths of our study is the use of the ImmunoCAP system, which remains the gold standard for in vitro measurement of allergen-specific IgE. The ImmunoCAP assay as a molecular diagnostic method for Fel d 1, involves relatively high costs, which may restrict its routine use in clinical or large-scale epidemiological settings, particularly in resource-limited environments. While the Fel d 1 testing is a more expensive option than the traditional methods, the clinical benefit of a highly accurate, predictive, and safer diagnostic tool often justifies the cost in complex cases or when considering long-term and costly immunotherapy in Egypt.

## Limitations

Despite the valuable insights provided by this study marking a significant milestone in Egyptian allergy diagnostics. Several limitations must be acknowledged. First, the sample size of 35 patients, though sufficient for demonstrating the diagnostic discrepancy between SPT and specific IgE levels, may clearly limit the generalizability of the findings to the entire Egyptian population and affects robustness and external validity of the results. Moreover, the observed correlations between Fel d 1-sIgE levels and disease severity should be interpreted as exploratory and hypothesis-generating, rather than definitive, due to limited statistical power. Second, the single-centre design and geographic focus with potential selection bias of patients could limit generalizability of results. Third, as a cross-sectional study, we could not track the longitudinal progression of sensitization or the long-term impact of AIT on Fel d 1-specific IgE levels. Logistical constraints and time limitations prevented the inclusion of longitudinal data or repeated measures that could have strengthened findings of this study. Resource constraints also rendered comparative analysis with various other diagnostic platforms not feasible. Fourth, the study focused specifically on Fel d 1, the major cat allergen, while other minor feline allergens like Fel d 2 and Fel d 4, were not tested due to financial constraints. This means we may have underestimated the total rate of ‘true’ cat allergy in our SPT-positive cohort, as some patients sensitised only to minor allergens would have been misclassified as SPT false-positives. CRD of minor feline allergens could have provided a more comprehensive picture of cross-reactivity with other furry animals. Additionally, environmental variables such as home ventilation, cleaning habits, and seasonal variation in allergen levels were not controlled, which may have influenced the measured allergen concentrations or participant responses. Besides IgE-mediated hypersensitivity reactions, patients may also produce non-IgE-mediated hypersensitivity reactions that may produce allergic diseases not detectable by the research of IgE^33^.

## Conclusion

This study addresses a critical gap in Middle Eastern allergy research. Our results highlight how crucial it is to integrate CRD, particularly Fel d 1-specific IgE testing by ImmunoCAP, compared to traditional extract-based methods into the standard diagnostic algorithm for Egyptian patients who show positive SPT but inconsistent clinical symptoms, to prevent unnecessary allergen avoidance and in polysensitised patients for enhancing diagnostic precision and detecting cross sensitization.

Introducing CRD into Egyptian allergy diagnostic protocols could help reduce misdiagnosis that potentially can help optimize immunotherapy selection and support personalized treatment strategies. The correlation between elevated Fel d 1-sIgE titers and increased disease severity warrants further large-scale validation. If confirmed, this biomarker could help clinicians prioritise those patients for early intervention strategies and innovative management, such as including anti-Fel d 1 IgY-enriched diets for cats, which have recently shown promising results ultimately improving outcomes for individuals with cat-induced respiratory allergies^31^.

## Data Availability

The datasets used and/or analysed during the current study are available from the corresponding author on reasonable request.
